# An Informant-Based Simple Questionnaire for Visuospatial Dysfunction Assessment in Dementia

**DOI:** 10.3389/fnins.2020.00044

**Published:** 2020-01-31

**Authors:** Ching-Tsu Wang, Guang-Uei Hung, Cheng-Yu Wei, Ray-Chang Tzeng, Pai-Yi Chiu

**Affiliations:** ^1^Department of Neurology, Tainan Municipal Hospital, Tainan, Taiwan; ^2^Department of Nuclear Medicine, Chang Bing Show Chwan Memorial Hospital, Changhua, Taiwan; ^3^Department of Exercise and Health Promotion, College of Education, Chinese Culture University, Taipei, Taiwan; ^4^Department of Neurology, Show Chwan Memorial Hospital, Changhua, Taiwan

**Keywords:** visuospatial dysfunction, Alzheimer’s disease, dementia with Lewy bodies, screening tools, cognitive abilities

## Abstract

**Objectives:**

Visuospatial dysfunction (VSD) is one of the most important symptoms for the diagnosis of dementia with Lewy bodies (DLB). The aim of this study was to validate a novel VSD questionnaire and determine the cutoff score for the screening for VSD in DLB.

**Methods:**

This is a retrospective analysis of data from a project of the History-based Artificial Intelligent Clinical Dementia Diagnostic System (HAICDDS). VSD of non-demented control (NDC), Alzheimer’s disease (AD), and DLB participants were analyzed and compared using the visuospatial questionnaire in the HAICDDS (HAI-VSQ), the Draw subscale in the Cognitive Abilities Screening Instrument (CASI-Draw), and the visuospatial subscale in Montreal Cognitive Assessment (MoCA-VS).

**Results:**

A total of 440 individuals were studied, including 154 NDC, 229 AD, and 57 DLB participants. Compared to NDC or AD participants, DLB participants showed a higher total score on HAI-VSQ after adjustment for age. Using HAI-VSQ, a cutoff score ≥ 2 was useful for the screening for VSD in DLB with a sensitivity of 0.77 and a specificity of 0.94. Compared with CASI-Draw or MoCA-VS, HAI-VSQ was least influenced by gender, age, and education and had the highest correlation with the sum of boxes of the Clinical Dementia Rating scale. After adjustment for age, education, gender, and global cognitive function, HAI-VSQ significantly discriminated DLB from AD and NDC whereas MoCA-VS or CASI-Draw did not.

**Conclusion:**

Our study showed that the newly designed simple questionnaire was a practical screening tool for VSD in DLB that can be applied in clinical practice as well as on a registration platform.

## Introduction

Visuospatial dysfunction (VSD) is a common clinical symptom used for the diagnosis of cognitive impairment or dementia due to Alzheimer’s disease (AD) (McKhann et al., 2011; Kim et al., 2017). Furthermore, VSD is the central symptom for the diagnosis of dementia with Lewy bodies (DLB) (McKeith et al., 2017). DLB is the second most-common type of degenerative dementia and previous studies have provided evidence of VSD in patients with DLB (McKeith et al., 2017). Previous studies assessing VSD in patients with dementia revealed several important clinical information with particular relevance for its connection to DLB. For example, salient and initial VSD are essential for the diagnosis of DLB (McKeith et al., 2017). Besides, well-formed, detailed, and complex visual hallucinations (VH) are among the core diagnostic features for DLB and this striking feature starts early in the disease (McKeith et al., 2017). The presence of VSD may also identify patients whose syndrome is driven by DLB rather than by AD pathology (Hamilton et al., 2012). The presence of early and severe VSD increases the likelihood that patients will develop prototypical DLB syndrome (Hamilton et al., 2012). In the early stages of dementia, VSD is more profound in DLB than in AD but memory retrieval deficit is more prominent in AD than in DLB (Yoshizawa et al., 2013). Studies of VSD mainly focused on the clinical performance of perception of locations or objects (Culham et al., 2006). Unlike these types of performance, visuomotor dysfunction manifests in goal-directed or visual-guided behavior and is also regarded as part of the visuospatial system of the brain (Culham et al., 2006). Visuomotor function is compromised in AD compared to normal elderly (Tippett et al., 2007; Galati et al., 2011; Hawkins and Sergio, 2014). Pathophysiological studies of visuomotor dysfunction revealed that reciprocal communication between hippocampal, parietal, and frontal brain regions play an important role in transforming visual-spatial information into goal-directed actions (Galati et al., 2011; Hawkins and Sergio, 2014). Disrupting these connections could affect the skills for activities of daily living (Hawkins and Sergio, 2014). Several studies using visuospatial/visuomotor function tests of the performance of visual recognition, visual discrimination, visual attention, or visuo-perceptive integration in DLB compared to AD revealed that these skills are impaired in DLB compared with AD (Oda et al., 2009; Yamaguchi et al., 2011; Li et al., 2014).

Based on these studies and diagnostic criteria, VSD including impairment of visuomotor skills is important for the diagnosis of dementia including AD and DLB. Therefore, a simple screening tool for VSD would be useful in a clinical setting but VSD assessment in common informant-based dementia assessment tools is still lacking and unable to satisfy the clinical requirements. For example, in the Clinical Dementia Rating (CDR) scale, evaluation of VSD is embedded in the domain of orientation and only a few questions address navigating function (Hamilton et al., 2012) but no question addresses visuomotor function. Impaired visuomotor skills are also important in other domains that are characteristic of DLB or posterior cortical atrophy (PCA) (McKhann et al., 2011; McKeith et al., 2017). Furthermore, frequently- used cognitive screening tests for dementia or cognitive impairment, for example, Mini-Mental Status Examination (MMSE), Cognitive Abilities Screening Instrument (CASI), and Montreal Cognitive Assessment (MoCA), also do not include visuomotor skills (Folstein et al., 1975; Teng et al., 1994; Chen et al., 2016).

To solve this problem, the initial aim of our study was to validate a novel VSD questionnaire that contained frequently-asked questions or common complaints of visuospatial and visuomotor symptoms obtained from caregivers or patients. In addition, we intended to use the simple questionnaire for investigating different presentations of VSD among the non-demented (ND) elderly, patients with neurodegenerative disorders including AD, DLB, or other disorders. Furthermore, during the consecutive data collection, the embedded auto-judgment program in the questionnaire continued to revise machine learning techniques to improve the ability of differential diagnosis of severity and subtypes of dementia.

## Materials And Methods

This is a sub-study of the History-based Artificial Intelligent Clinical Dementia Diagnostic System (HAICDDS) project which is currently used as a registration platform in the Show Chwan Health System. Before the starting of the study, twenty-six participants with their informants were interviewed by neuropsychologists from 3 centers of the health system and the reproducibility was investigated using the interrater reliability analysis. The results revealed a high intra-class correlation coefficient of 0.830. The detailed procedure of this project was described in our previous reports (Lin et al., 2018; Chiu et al., 2019a, b). In this study, we analyzed the data of individuals with normal cognition (NC), mild cognitive impairment (MCI), and dementia due to DLB or AD.

### Diagnosis of AD or DLB

The diagnosis of DLB was made according to the revised consensus criteria for probable DLB developed by the fourth report of the DLB consortium (McKeith et al., 2017). According to these criteria, at least two of the following core features including fluctuation of cognition, VH, parkinsonism, and REM sleep behavior disorder (RBD) or one core features plus at least one indicative biomarker including abnormal dopamine transporter imaging (DaTabN), abnormal ^123^I-metaiodobenzylguanidine (MIBG), and REM sleep without atonia (RSWA) were necessary for the diagnosis of probable DLB. AD patients were diagnosed according to the criteria for probable AD developed by the National Institute on Aging and Alzheimer’s Association Workgroup (NIA-AA) (Kim et al., 2017).

### Diagnosis of Non-demented Control (NDC) or Different Stages of Dementia

For the diagnosis of NDC, patients should have NC or MCI. NC was diagnosed with a global CDR (Morris, 1993) score of 0. MCI was diagnosed based on the criteria for MCI of the National Institute on Aging and Alzheimer’s Association Workgroup on 2011 (Albert et al., 2011) as a change in cognition with impairment in one or more cognitive domains but no evidence of impairment in social or occupational functioning with a CDR score of 0.5 and the sum of boxes of CDR (CDR-SB) 0.5–4.0 (O’Bryant et al., 2008). The diagnosis of dementia was made according to the criteria for dementia developed by the NIA-AA (Kim et al., 2017). Participants with dementia had impairments in two or more cognitive domains as well as a decline in daily functions (at least one of the domains of community affairs, home hobbies, and personal care with a CDR ≥ 0.5). Dementia severity was defined by a global CDR scale. A global CDR score of 0.5, 1, 2, and 3 was defined as very mild, mild, moderate, and severe dementia, respectively (Morris, 1993).

### Procedure of the Study

This is a retrospective analysis of data from the HAICDDS which is currently applied in three centers in Taiwan (two in central Taiwan and one in southern Taiwan). In the database, daily function was assessed using the Instrumental Activities of Daily Living (IADL) Scale (Lawton and Brody, 1969). Cognitive function was assessed using the Cognitive Abilities Screening Instrument (CASI) (Teng et al., 1994) and the Montreal Cognitive Assessment (MoCA) (Chen et al., 2016). Cognitive tests for all patients were performed by trained neuropsychologists. VSD was assessed using the visuospatial subscale of CASI (CASI-Draw, total score 0–10), of MoCA (MoCA-VS, total score 0–5), and of the HAICDDS (HAI-VSQ, total score 0–12) which includes 7 visuospatial/visuomotor function questions (The original Chinese version of the questionnaire with tentative English translation is shown in Appendix Table A1). VSD of NDC, AD, and DLB were analyzed and compared. In performing HAICDDS, informants of the participants were interviewed by a well-trained neuropsychologist and were requested to fill out the original structured questionnaire to determine the severity of dementia or cognitive impairment.

### Statistics

The Chinese version of SPSS 22.0 for Windows (IBM, SPSS Inc., Chicago) was used for statistical analyses. For the determination of cut-off score for the differentiation from DLB to NCD, we want to maximize both the sensitivity and specificity therefore, the Youden’s index was applied, which is maximum = sensitivity + specificity − 1. Comparisons of demographic data, neuropsychological tests, sum of boxes of CDR (CDR-SB), IADL, MoCA, MoCA-VS, CASI, CASI-Draw, HAI-VSQ, and sum of score of the Neuropsychiatric Inventory (NPI-sum) (Cummings, 1988) were compared between the different groups and analyzed using independent t-tests or one-way ANOVA with either Bonferroni or Dunnett T3 *post hoc* analysis according to the homogeneity of variance. Gender and DLB clinical features (fluctuation, VH, RBD, Parkinsonism, and abnormal dopamine transporter imaging) (McKeith et al., 2017) were analyzed with the chi-square test. Multivariable risk estimates (OR) for each question in HAI-VSQ, CASI-Draw, and MoCA-VSQ were adjusted for age, gender, education, and cognitive function and compared between AD/NDC, DLB/NDC, and DLB/AD groups. Pearson correlation coefficients were derived between age, education, gender, CDR-SB, IADL, CASI, MoCA, and NPI of the different diagnostic tools for VSD.

### Ethical Consideration

The participants were selected from a register-based database of Show Chwan Health System. The study design was retrospective and the data were analyzed anonymously. The Committee for Medical Research Ethics of Show Chwan Memorial Hospital reviewed the project and the Data Inspectorate approved the study.

## Results

**FIGURE 1:**
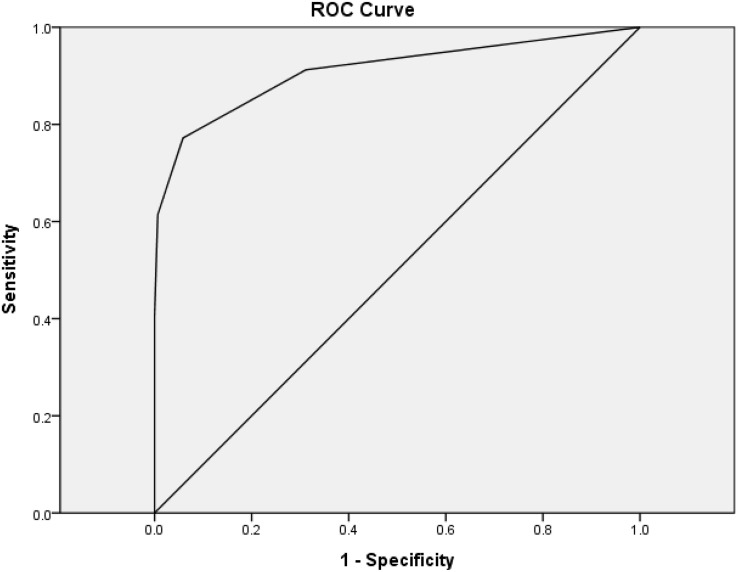
Receiver–operating characteristic curves (ROC) of the HAI-VSQ for the discrimination of VSD in DLB and NDC. A cutoff score ≥ 2 was useful with a sensitivity of 0.77, a specificity of 0.94, and an AUC of 0.91.

A total of 440 individuals were studied, including 154 NDC, 229 AD, and 57 DLB participants. One or more visuospatial symptoms were reported in 31.2% of NC, 81.7% of AD, and 91.2% of DLB participants. Mean age of the NDC group (71.3 ± 9.2) was significantly smaller than those of DLB (81.3 ± 7.0) or AD (80.2 ± 7.2) using one-way ANOVA (*F* = 65.58; *p* < 0.001). After adjustment for age, the dementia groups showed impaired responses to all questions compared to the NDC group (all *p* < 0.001). Compared to NDC (0.4 ± 0.6) or AD (2.0 ± 2.2), the DLB (3.3 ± 2.2) group showed significantly increased total score of the HAI-VSQ after adjustment for age (both *p* < 0.001). Using HAI-VSQ, a cutoff score ≥ 2 was useful for the discrimination of VSD in DLB and NDC with a sensitivity of 0.77, a specificity of 0.94, and an AUC of 0.91 (Figure 1).

Additionally, the DLB group demonstrated higher CDR-SB, NPI, and Lewy body clinical features, including fluctuation of cognition, VH, Parkinsonism, and RBD after adjustment for age (all *p* < 0.001). The DLB group also demonstrated significant lower IADL, CASI, and MoCA compared to NDC or AD participants after adjustment for age (all *p* < 0.001). Compared to NDC participants, AD patients showed significantly higher CDR-SB, were proportionally more often female, had a significantly higher total score of the HAI-VSQ, NPI, and significantly higher fluctuation after adjustment for age (all *p* < 0.001). The AD patients also demonstrated lower education, IADL, MoCA, and CASI compared to NDC participants (Table 1).

**TABLE 1:** Comparison of demographic data among the NDC (*N* = 154), AD (*N* = 229), and DLB (*N* = 57) participants.

	NDC mean (*SD*)	AD mean (*SD*)	DLB mean (*SD*)	AD vs. NDC OR, *p*-value	DLB vs. NDC *p*-value	DLB vs. AD *p*-value
Age, year	71.3 (9.2)	80.2 (7.2)	81.3 (7.0)	NA	NA	NA
CDR-SB	0.8 (0.7)	7.0 (4.6)	10.3 (5.3)	11.25, < 0.001	4.78, < 0.001	1.15, < 0.001
Female, N (%)	81 (52.6)	151 (65.9)	32 (56.1)	1.88, 0.010	0.92, NS	0.67, NS
Education	7.1 (5.1)	4.1 (4.4)	3.2 (3.7)	0.93, 0.003	0.90, 0.027	0.95, NS
HAI-VSQ	0.4 (0.6)	2.0 (2.2)	3.3 (2.2)	3.68, < 0.001	5.62, < 0.001	1.26, < 0.001
IADL	7.5 (1.0)	2.3 (2.6)	1.2 (2.1)	0.31, < 0.001	0.37, < 0.001	0.78, 0.003
MoCA	19.1 (6.1)	6.8 (5.1)	5.8 (4.8)	0.75, < 0.001	0.70, < 0.001	0.95, NS
CASI	79.4 (12.2)	41.8 (22.8)	39.7 (21.8)	0.90, < 0.001	0.89, < 0.001	0.99, NS
NPI	3.1 (5.0)	5.4 (7.7)	16.0 (16.4)	1.09, < 0.001	1.23, < 0.001	1.10, < 0.001
Fluctuation, N (%)	2 (1.3)	36 (15.7)	37 (64.9)	12.87, < 0.001	135.9, < 0.001	9.93, < 0.001
VH, N (%)	4 (2.6)	12 (5.2)	15 (26.3)	1.69, NS	17.9, < 0.001	6.64, < 0.001
Parkinsonism, N (%)	18 (11.8)	26 (11.4)	51 (89.5)	0.68, NS	41.86, < 0.001	66.64, < 0.001
RBD, N (%)	17 (11.0)	9 (3.9)	22 (38.6)	0.34, 0.023	7.89, < 0.001	17.47, < 0.001
DaTabN*, N (%)	5 (35.7)	3 (27.3)	13 (72.2)	0.64, NS	3.84, NS	10.8, 0.018
Informer age, year	61.0 (12.6)	54.1 (10.2)	56.8 (12.4)	0.94, < 0.001	0.96, 0.035	1.02, NS
Informer education, year	10.0 (4.4)	12.1 (3.3)	11.2 (3.5)	1.12, 0.011	1.07, NS	0.91, NS

Odds ratio (OR) was Adjusted for Age. NDC, Non-demented control, including normal cognition and mild cognitive impairment; AD, Alzheimer’s disease; DLB, dementia with Lewy bodies; NA, Not applicable; NS, Non-significance; CDR-SB, Sum of boxes of the Clinical Dementia Rating Scale; HAI-VSQ, Visuospatial function questionnaire in the History-based Artificial Intelligent Clinical Dementia Diagnostic System (HAICDDS); IADL, Instrumental Activities of Daily Living; MoCA, Montreal Cognitive Assessment; CASI, Cognitive Abilities Screening Instrument; NPI, Neuropsychiatric Inventory; VH, Visual hallucinations; RBD, REM sleep behavior disorder; DaTabN*, Abnormal dopamine transporter imaging among eight ND, 12 AD, and 17 DLB participants.

Pearson correlation coefficients between age, education, gender, CDR-SB, IADL, CASI, MoCA, and NPI of different diagnostic tools for VSD are summarized in Table 2. The HAI-VSQ had weak to moderate correlation with MoCA-VS (*r* = -0.380, *p* < 0.001) or CASI-Draw (*r* = -0.467, *p* < 0.001). Furthermore, except for the non-correlation between HAI-VSQ with gender (*r* = -0.026, *p* < 0.341), other parameters were significantly correlated. In contrast to HAI-VSQ, MoCA-VS (*r* = 0.234, *p* < 0.001) and CASI-Draw (*r* = 0.187, *p* < 0.001) were weakly correlated to gender. Compared to MoCA-VS or CASI-Draw, HAI-VSQ had the lowest correlation with age or education and the highest correlation with CDR-SB.

**TABLE 2:** Point-Biserial correlation coefficients between age, education, gender, CDR-SB, IADL, CASI, MoCA, and NPI of different diagnostic tools for visuospatial dysfunction among all participants in NDC, AD, and DLB Groups.

	Age	Education	Gender	CDR-SB	IADL	MoCA	CASI	NPI	HAI-VSQ	MoCA-VS	CASI-Draw
HAI-VSQ	*r* = 0.330 *p* < 0.001	*r* = −0.112 *p* = 0.019	*r* = −0.026 *p* = 0.341	*r* = 0.825 *p* < 0.001	*r* = −0.602 *p* < 0.001	*r* = −0.552 *p* < 0.001	*r* = −0.683 *p* < 0.001	*r* = 0.235 *p* < 0.001		*r* = −0.380 *p* < 0.001	*r* = −0.467 *p* < 0.001
MoCA-VS	*r* = −0.550 *p* < 0.001	*r* = 0.584 *p* < 0.001	*r* = 0.234 *p* < 0.001	*r* = −0.537 *p* < 0.001	*r* = 0.598 *p* < 0.001	*r* = 0.842 *p* < 0.001	*r* = 0.715 *p* < 0.001	*r* = −0.131 *p* = 0.006	*r* = −0.380 *p* < 0.001		*r* = 0.599 *p* < 0.001
CASI-Draw	*r* = −0.396 *p* < 0.001	*r* = 0.424 *p* < 0.001	*r* = 0.187 *p* < 0.001	*r* = −0.612 *p* < 0.001	*r* = 0.565 *p* < 0.001	*r* = 0.687 *p* < 0.001	*r* = 0.786 *p* < 0.001	*r* = −0.140 *p* = 0.003	*r* = −0.467 *p* < 0.001	*r* = 0.599 *p* < 0.001	

**CDR-SB, Sum of boxes of the Clinical Dementia Rating Scale; IADL, Instrumental Activities of Daily Living; MoCA, Montreal Cognitive Assessment; CASI, Cognitive Abilities Screening Instrument; NPI, Neuropsychiatric Inventory; HAI-VSQ, Sum score of the visuospatial questionnaire in History-based Artificial Intelligent Clinical Dementia Diagnostic System (HAICDDS); MoCA-VS, Visuospatial domain in MoCA; CASI-Draw, Visuospatial domain in CASI.**

The comparison of visuospatial subscales in HAI-VSQ, MoCA-VS, and CASI-Draw among NDC, AD, and DLB, the odds ratio (OR) adjusted for age, education, gender, and the cognitive state by CASI total score are summarized in Table 3. The HAI-VSQ significantly discriminated DLB from AD or NDC whereas MoCA-VS or CASI-Draw did not.

**TABLE 3:** Comparison of visuospatial subscales in HAICDDS (HAI-VSQ), MoCA (MoCA-VS), and CASI (CASI-Draw) among NDC (*N* = 154), AD (*N* = 229), and DLB (*N* = 57) participants.

	NDC mean (SD)	AD mean (SD)	DLB mean (SD)	AD vs. NDC OR, *p*-value	DLB vs. NDC OR, *p*-value	DLB vs. AD OR, *p*-value
HAI-VSQ	0.4 (0.6)	2.0 (2.2)	3.3 (2.2)	2.79, < 0.001	4.28, < 0.001	1.48, < 0.001
MoCA-VS	2.5 (1.6)	0.5 (1.0)	0.4 (0.7)	0.81, NS	0.70, NS	0.96, NS
CASI-Draw	8.6 (2.5)	4.9 (4.1)	4.3 (3.5)	1.25, 0.003	1.03, NS	1.02, NS

**Odds Ratio (OR) was Adjusted for Age, Education, Gender, and Cognitive State by CASI Total Score. *NDC, Non-demented control; AD, Alzheimer’s disease; DLB, dementia with Lewy bodies; NA, Not applicable; NS, Non-significance; HAI-VSQ, Visuospatial function questionnaire in History-based Artificial Intelligent Clinical Dementia Diagnostic System (HAICDDS); MoCA-VS, Visuospatial domain in MoCA; CASI-Draw, Visuospatial domain in CASI.***

**FIGURE 2:**
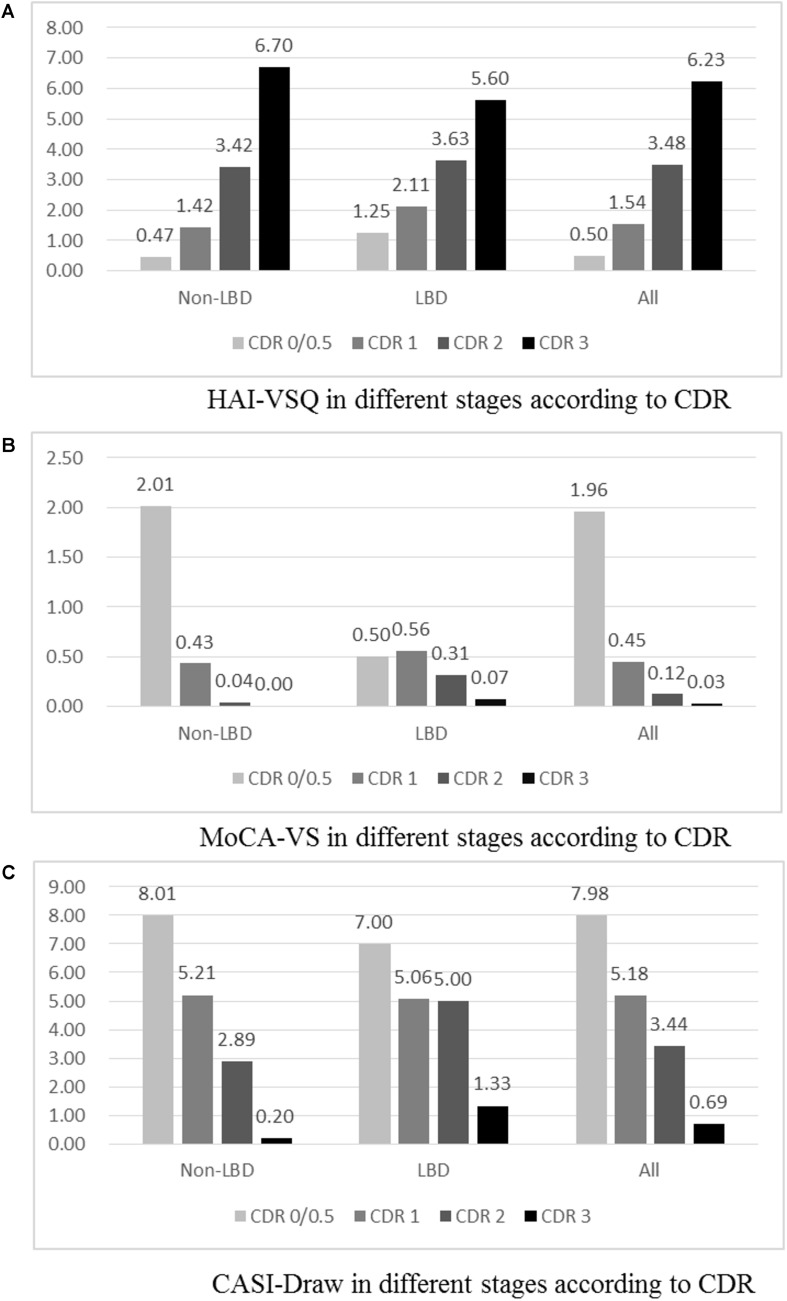
Visuospatial subscales of HAICDDS (HAI-VSQ, **A**), MoCA (MoCA-VS, **B**), and CASI (CASI-Draw, **C**) in different stages of dementia with Lewy bodies (DLB), non-DLB, and all participants. Participants with CDR 0 were classified only in the non-LBD group and all participants. **(A)** HAI-VSQ in different stages according to CDR. **(B)** MoCA-VS in different stages according to CDR. (1c) CASI-Draw in different stages according to CDR.

Visuospatial subscales of HAI-VSQ (A), MoCA-VS (B), and CASI-Draw (C) in different stages of dementia with Lewy bodies (DLB) and non-DLB are summarized in Figure 2. Among all participants, significantly increased HAI-VSQ (all *p* < 0.001) and decreased CASI-Draw (all *p* < 0.05) were noted as the severity of dementia increased. MoCA-VS was different in the CDR 0/0.5 stage compared to other stages (all *p* < 0.001). Among DLB participants, HAI-VSQ showed significant differences in CDR 3 vs. CDR 2, CDR 3 vs. CDR 1, CDR 3 vs. CDR 0/0.5, and CDR 2 vs. CDR 0/0.5. CASI-Draw showed significant differences in CDR 0/0.5 vs. CDR 1, CDR 2, and CDR 3. MoCA-VS did not differentiate between any two stages according to CDR. Among non-DLB participants, significantly increased HAI-VSQ (all *p* < 0.001) and decreased CASI-Draw (all *p* < 0.05) were noted as the severity of dementia increased. Except for CDR 2 vs. CDR 3, significantly decreased MoCA-VS (all *p* < 0.005) was noted as the severity of dementia increased.

## Discussion

This study was a sub-study of the HAICDDS project, analyzing and comparing data between NDC, AD, and DLB groups with two main results. First, using HAI-VSQ, a cutoff score ≥ 2 discriminates VSD in DLB from NDC with high sensitivity (0.77), specificity (0.94), and AUC (0.91). To provide more objective evidence, we analyzed the correlation of the HAI-VSQ with dopamine transporter imaging among NDC and DLB groups and the result showed a high negative correlation of striatal background ratio (SBR) of dopamine transporter imaging with the HAI-VSQ with correlation coefficient -0.571 and *p* < 0.001. These findings have provided additional evidence that the HAI-VSQ has high correlation with DLB because of abnormal dopamine transporter imaging being the indicative biomarker for the diagnosis of DLB. Therefore, we provided a simple tool that can help clinicians to detect DLB more easily at the bedside or in clinics. Additionally, HAI-VSQ is probably the first informant-based VSD questionnaire that includes not only visuospatial but also visuomotor questions. We found more severe VSD according to the questionnaire in patients with DLB compared to NDC or AD. In this study, one or more visuospatial or visuomotor symptoms were reported in 31.2% of NDC, and 81.7% of AD and 91.2% of DLB patients. In mild stages, symptoms were reported in 74.4% of AD and 80.8% of DLB patients in CDR = 0.5 or 1. These results are consistent with previous studies on VSD that showed common and early symptoms in AD as well as in DLB (Culham et al., 2006; Tippett et al., 2007; Oda et al., 2009; Galati et al., 2011; Yamaguchi et al., 2011; Hamilton et al., 2012; Yoshizawa et al., 2013; Hawkins and Sergio, 2014; Li et al., 2014). More severe VSD in DLB according to the HAI-VSQ compared to AD is also consistent with results from previous studies (Oda et al., 2009; Yamaguchi et al., 2011; Li et al., 2014).

Second, compared to MoCA-VS or CASI-Draw, HAI-VSQ had no correlation with gender and the lowest correlation with age and education. We considered this an important result because the current frequently-used dementia screening tools such as MoCA or CASI are too sensitive to age, gender, culture, and education. Therefore, a large variety of cut-off scores and adjustments are necessary for the screening of dementia or cognitive impairment when using these tools (Lin et al., 2002; Nasreddine et al., 2005; Chen et al., 2016). Furthermore, HAI-VSQ had the highest correlation with CDR-SB which had the highest correlation with dementia severity compared to the MoCA-VS or CASI-Draw scales.

In addition to above-mentioned findings, after adjustment for age, education, gender, and cognitive state by CASI total score, HAI-VSQ was significantly different in DLB patient than in NDC (OR = 4.28, *p* < 0.001) or AD patients (OR = 1.48, *p* < 0.001), and between AD patients and NDC participants (OR = 2.79, *p* < 0.001). MoCA-VS or CASI-Draw showed no significant differences. This finding underlines the clinical applicability of the HAI-VSQ for the discrimination of DLB from AD or NDC and of AD from NDC participants. This also indicates that the information acquired from caregivers may be more useful or at least as useful as the cognitive performance of patients because the caregivers directly face the caring problems which might result in a higher impact of VSD on them.

Finally, we want to address the important issue that commonly-used informant-based questionnaires for the screening of dementia or cognitive impairment from normal elderly including CDR, AD8, or IQCODE are lacking or have only a few questions regarding VSD (Jorm et al., 1991; Morris, 1993; Galvin et al., 2005; Razavi et al., 2014). We are providing a simple informant-based visuospatial questionnaire for the clinical assessment of individuals with dementia. The purpose of our study was not using a cut-off score for the discrimination of patients with language dysfunction from normal people. Instead, we want to provide an easy way for clinicians to be aware of the visuospatial as well as visuomotor problems of patients with dementia due to AD or DLB.

There are several limitations to this study. First, the questionnaire is an original Taiwanese version. More precise and colloquial translations will be necessary when translating the questionnaire to other language versions although we have preliminarily translated the questionnaire to English. Second, our study was conducted in only three centers in Taiwan and the questionnaire contained only seven questions. The findings of different presentations of VSD may not be generalizable to all individuals with NDC, AD, or DLB. Third, the comparison among different groups in our study was retrospective and cross-sectional. Therefore, a causal relationship between VSD and the underlying pathophysiologies of AD or DLB could not be investigated.

## Conclusion

In conclusion, our study showed that similar to our recently published language questionnaire (HAICDDS-Language) (Lin et al., 2018), the informant-based simple questionnaire was a practical screening tool and was more applicable than the visuospatial subscale of MoCA or CASI for the discrimination of NDC, AD, and DLB. We intend to design and validate several dementia-related simple questionnaires and hope that these rapid screening tools can be applied in clinical practice as well as in a registration platform for the screening of VSD as well as other cognitive dysfunctions. A further goal is to implement machine learning techniques to improve the accuracy and efficiency of these questionnaires.

## Data Availability Statement

The raw data supporting the conclusions of this article will be made available by the authors, without undue reservation, to any qualified researcher.

## Ethics Statement

The studies involving human participants were reviewed and approved by the Show Chwan Memorial Hospital. Written informed consent for participation was not required for this study in accordance with the national legislation and the institutional requirements.

## Author Contributions

C-TW undertook the literature search and data analysis, edited the Author Contributions, and was mainly responsible for the revisions and drafts of the manuscript. P-YC participated in the data analysis and contributed to the revisions and final draft of the manuscript. G-UH and R-CT undertook the literature search and contributed to revisions. C-YW contributed to revisions of the manuscript.

## Conflict of Interest

P-YC’s work has been partly supported by the Show Chwan Memorial Hospital. The remaining authors declare that the research was conducted in the absence of any commercial or financial relationships that could be construed as a potential conflict of interest.

## Appendix

**TABLE A1:** Composition of the visuospatial questionnaire in HAICDDS (HAI-VSQ).

VS1	方向感變差了嗎?	□不會	□會	
	Does he/she have trouble finding directions?	□ No	□ Yes	
VS2	在熟悉的環境（例如：住家附近）會迷路嗎?	□ 不會	□ 會	
	Does he/she get lost in familiar surroundings, for example, the neighborhood?	□ No	□ Yes	
VS3	會搞不清楚自己在哪裏嗎?	□ 不會	□ 偶而	□ 常常
	Does he/she have trouble locating himself/herself?	□ No	□ Occasionally	□ Often
VS4	會「常常｣認錯人，例如：把兒子當成丈夫，把女兒當成姊妹嗎?	□ 不會	□ 偶而	□ 常常
	Does he/she often recognize the wrong person, for example, recognizing son as husband or daughter as sister?	□ No	□ Occasionally	□ Often
VS5	□走路、騎車或是開車的時候沒辦法直直走，常常偏到旁邊去嗎?	□ 不會	□ 偶而	□ 常常
	Does he/she often deviate to one side during walking, riding, or driving on the road?	□ No	□ Occasionally	□ Often
VS6	沒辦法順利地開門，好像找不太到鑰匙孔或門把嗎?	□ 不會	□ 偶而	□ 常常
	Does he/she have difficulties in finding the keyhole or doorknob for opening the door?	□ No	□ Occasionally	□ Often
VS7	閱讀或是寫字變得困難嗎?	□ 不會	□ 偶而	□ 常常
	Does he/she have difficulties in reading or writing?	□ No	□ Occasionally	□ Often

## References

[B1] Albert M. S. DeKosky S. T. Dickson D. Dubois B. Feldman H. H. Fox N. C. (2011). The diagnosis of mild cognitive impairment due to Alzheimer’s disease: recommendations from the National Institute on Aging-Alzheimer’s Association workgroups on diagnostic guidelines for Alzheimer’s disease. *Alzheimers Dement.* 7 270–279. 10.1016/j.jalz.2011.03.008 21514249 PMC3312027

[B2] Chen K. L. Xu Y. Chu A. Q. Ding D. Liang X. N. Nasreddine Z. S. (2016). Validation of the Chinese version of Montreal cognitive assessment basic for screening mild cognitive impairment. *J. Am. Geriatr. Soc.* 64 e285–e290. 10.1111/jgs.14530 27996103

[B3] Chiu P. Y. Tang H. Wei C. Y. Zhang C. Hung G. U. Zhou W. (2019a). NMD-12: a new machine-learning derived screening instrument to detect mild cognitive impairment and dementia. *PLoS One* 14:e0213430. 10.1371/journal.pone.0213430 30849106 PMC6407752

[B4] Chiu P.-Y. Wei C.-Y. Hung G.-U. (2019b). Preliminary study of the history-based artificial intelligent clinical dementia diagnostic system. *Show Chwan Med. J.* 18 18–27. 10.3966/156104972019061801003

[B5] Culham J. C. Cavina-Pratesi C. Singhal A. (2006). The role of parietal cortex in visuomotor control: what have we learned from neuroimaging? *Neuropsychologia* 44 2668–2684. 10.1016/j.neuropsychologia.2005.11.003 16337974

[B6] Cummings J. L. (1988). Intellectual impairment in Parkinson’s disease: clinical, pathologic, and biochemical correlates. *J. Geriatr. Psychiatry Neurol.* 1 24–36. 10.1177/089198878800100106 2908099

[B7] Folstein M. F. Folstein S. E. McHugh P. R. (1975). “Mini-mental state”. A practical method for grading the cognitive state of patients for the clinician. *J. Psychiatr. Res.* 12 189–198. 1202204 10.1016/0022-3956(75)90026-6

[B8] Galati G. Committeri G. Pitzalis S. Pelle G. Patria F. Fattori P. (2011). Intentional signals during saccadic and reaching delays in the human posterior parietal cortex. *Eur. J. Neurosci.* 34 1871–1885. 10.1111/j.1460-9568.2011.07885.x 22017280

[B9] Galvin J. E. Roe C. M. Powlishta K. K. Coats M. A. Muich S. J. Grant E. (2005). The AD8: a brief informant interview to detect dementia. *Neurology* 65 559–564. 10.1212/01.wnl.0000172958.95282.2a 16116116

[B10] Hamilton J. M. Landy K. M. Salmon D. P. Hansen L. A. Masliah E. Galasko D. (2012). Early visuospatial deficits predict the occurrence of visual hallucinations in autopsy-confirmed dementia with Lewy bodies. *Am. J. Geriatr. Psychiatry* 20 773–781. 10.1097/JGP.0b013e31823033bc 21997600 PMC3260388

[B11] Hawkins K. M. Sergio L. E. (2014). Visuomotor impairments in older adults at increased Alzheimer’s disease risk. *J. Alzheimers Dis.* 42 607–621. 10.3233/jad-140051 24919768

[B12] Jorm A. F. Scott R. Cullen J. S. MacKinnon A. J. (1991). Performance of the informant questionnaire on cognitive decline in the elderly (IQCODE) as a screening test for dementia. *Psychol. Med.* 21 785–790. 10.1017/s0033291700022418 1946866

[B13] Kim J. Na H. K. Byun J. Shin J. Kim S. Lee B. H. (2017). Tracking cognitive decline in amnestic mild cognitive impairment and early-stage alzheimer dementia: mini-mental state examination versus neuropsychological battery. *Dement. Geriatr. Cogn. Disord.* 44 105–117. 10.1159/000478520 28768247

[B14] Lawton M. P. Brody E. M. (1969). Assessment of older people: self-maintaining and instrumental activities of daily living. *Gerontologist* 9 179–186. 10.1093/geront/9.3_part_1.179 5349366

[B15] Li X. Rastogi P. Gibbons J. A. Chaudhury S. (2014). Visuo-cognitive skill deficits in Alzheimer’s disease and Lewy body disease: a comparative analysis. *Ann. Indian Acad. Neurol.* 17 12–18. 10.4103/0972-2327.128530 24753653 PMC3992750

[B16] Lin C. M. Hung G. U. Wei C. Y. Tzeng R. C. Chiu P. Y. (2018). An informant-based simple questionnaire for language assessment in neurodegenerative disorders. *Dement. Geriatr. Cogn. Disord.* 46 207–216. 10.1159/000493540 30336484

[B17] Lin K. N. Wang P. N. Liu C. Y. Chen W. T. Lee Y. C. Liu H. C. (2002). Cutoff scores of the cognitive abilities screening instrument, Chinese version in screening of dementia. *Dement. Geriatr. Cogn. Disord.* 14 176–182. 10.1159/000066024 12411759

[B18] McKeith I. G. Boeve B. F. Dickson D. W. Halliday G. Taylor J. P. Weintraub D. (2017). Diagnosis and management of dementia with Lewy bodies: fourth consensus report of the DLB consortium. *Neurology* 89 88–100. 10.1212/wnl.0000000000004058 28592453 PMC5496518

[B19] McKhann G. M. Knopman D. S. Chertkow H. Hyman B. T. Jack C. R. Jr. Kawas C. H. (2011). The diagnosis of dementia due to Alzheimer’s disease: recommendations from the National Institute on Aging-Alzheimer’s Association workgroups on diagnostic guidelines for Alzheimer’s disease. *Alzheimers Dement.* 7 263–269. 10.1016/j.jalz.2011.03.005 21514250 PMC3312024

[B20] Morris J. C. (1993). The clinical dementia rating (CDR): current version and scoring rules. *Neurology* 43 2412–2414. 10.1212/wnl.43.11.2412-a 8232972

[B21] Nasreddine Z. S. Phillips N. A. Bedirian V. Charbonneau S. Whitehead V. Collin I. (2005). The Montreal cognitive assessment, MoCA: a brief screening tool for mild cognitive impairment. *J. Am. Geriatr. Soc.* 53 695–699. 10.1111/j.1532-5415.2005.53221.x 15817019

[B22] O’Bryant S. E. Waring S. C. Cullum C. M. Hall J. Lacritz L. Massman P. J. (2008). Staging dementia using clinical dementia rating scale sum of boxes scores: a Texas Alzheimer’s research consortium study. *Arch. Neurol.* 65 1091–1095. 10.1001/archneur.65.8.1091 18695059 PMC3409562

[B23] Oda H. Yamamoto Y. Maeda K. (2009). The neuropsychological profile in dementia with Lewy bodies and Alzheimer’s disease. *Int. J. Geriatr. Psychiatry* 24 125–131. 10.1002/gps.2078 18615776

[B24] Razavi M. Tolea M. I. Margrett J. Martin P. Oakland A. Tscholl D. W. (2014). Comparison of 2 informant questionnaire screening tools for dementia and mild cognitive impairment: AD8 and IQCODE. *Alzheimer Dis. Assoc. Disord.* 28 156–161. 10.1097/wad.0000000000000008 24113559 PMC3981951

[B25] Teng E. L. Hasegawa K. Homma A. Imai Y. Larson E. Graves A. (1994). The cognitive abilities screening instrument (CASI): a practical test for cross-cultural epidemiological studies of dementia. *Int. Psychogeriatr.* 6 45–58 discussion 62. 8054493 10.1017/s1041610294001602

[B26] Tippett W. J. Krajewski A. Sergio L. E. (2007). Visuomotor integration is compromised in Alzheimer’s disease patients reaching for remembered targets. *Eur. Neurol.* 58 1–11. 10.1159/000102160 17483579

[B27] Yamaguchi H. Takahashi S. Kosaka K. Okamoto K. Yamazaki T. Ikeda M. (2011). Yamaguchi fox-pigeon imitation test (YFPIT) for dementia in clinical practice. *Psychogeriatrics* 11 221–226. 10.1111/j.1479-8301.2011.00373.x 22151241

[B28] Yoshizawa H. Vonsattel J. P. Honig L. S. (2013). Early neuropsychological discriminants for Lewy body disease: an autopsy series. *J. Neurol. Neurosurg. Psychiatry* 84 1326–1330. 10.1136/jnnp-2012-304381 23308020

